# The Optimal Management of Patients with Prostate Cancer with Oligometastatic Disease and Low Metastatic Burden in the PSMA-PET Era

**DOI:** 10.3390/cancers18030450

**Published:** 2026-01-30

**Authors:** Menal Bhandari, Isaac Lasko, Jacob Pozin, Michael Chang, Hann-Hsiang Chao, Elizabeth Henry, Nicholas G. Nickols, Timothy Ritter, Joseph K. Salama, Abhishek A. Solanki

**Affiliations:** 1 Department of Radiation Oncology, Stritch School of Medicine, Loyola University Chicago, Maywood, IL 60153, USA; menal.bhandari@luhs.org (M.B.); isaac.lasko@luhs.org (I.L.); 2 Department of Internal Medicine, Stritch School of Medicine, Loyola University Chicago, Maywood, IL 60153, USA; jacob.pozin@luhs.org (J.P.); ehenry@luc.edu (E.H.); 3 Radiation Oncology Service, Hunter Holmes McGuire VA Medical Center, Richmond, VA 23249, USA; michael.chang3@va.gov (M.C.); hann-hsiang.chao@va.gov (H.-H.C.); timothy.ritter@va.gov (T.R.); 4 Department of Radiation Oncology, VA Greater Los Angeles Healthcare System, Los Angeles, CA 90073, USA; nicholas.nickols@va.gov; 5 Department of Radiation Oncology, Duke University Medical Center, Durham, NC 27710, USA; joseph.salama@duke.edu

**Keywords:** metastatic prostate cancer, low metastatic burden, oligometastasis, metastasis-directed therapy

## Abstract

Historically, systemic therapy has been the primary treatment for metastatic prostate cancer (MPC), with radiotherapy and surgery reserved for palliation. The recent literature suggests that adding local therapy (i.e., radiotherapy or surgery) to systemic therapy may improve survival for MPC patients with low metastatic burden (LMB). While some evidence supports the use of early intervention with local therapy targeting both the primary tumor and limited metastatic sites, the definition of LMB disease requires further clarification. Prostate-specific membrane antigen (PSMA) positron emission tomography (PET) scans play a vital role in staging MPC because they offer superior sensitivity and specificity compared to conventional imaging. PSMA PET thus improves patient selection and helps direct treatment planning. Local therapy in MPC can be separated into the treatment of primary and metastatic tumors. Furthermore, treatment of both the primary tumor and metastases can be managed using either radiotherapy or surgical intervention. Studies exploring the use of local therapy for both the primary tumor and oligometastatic sites have demonstrated promising clinical outcomes in patients with LMB or oligometastatic disease. This review provides a detailed description of the current optimal management of patients with metastatic prostate cancer with limited disease.

## Simple Summary

Historically, metastatic prostate cancer has been treated primarily with medication that circulates throughout the body (systemic therapy), and radiation or surgery were mostly used for symptom relief. However, recent research suggests that adding targeted treatment with radiation or surgery to the prostate itself and to the metastatic sites might improve survival for patients whose cancer has metastasized to only a limited number of areas. Integration of functional imaging modalities is now essential in this field. Research continues to progress in this direction to optimize treatment.

## 1. Introduction

In 2025, about 313,780 new prostate cancer cases were predicted, with a 4.5% increase in incidence every year since 2014 for advanced or metastatic disease [1]. Age-adjusted prostate cancer mortality for American men over 45 has decreased substantially from 1999 to 2013, but since then, overall mortality from prostate cancer actually began to rise. This recent increase in total prostate cancer-related deaths presents a major public health challenge, compounded by persistent disparities; non-Hispanic Black men experience mortality rates more than double those of non-Hispanic White men, with further inequities driven by age, geography, and levels of urbanization [2].

The prognosis and management of metastatic prostate cancer (MPC) are based on the type of imaging used to identify metastases, the extent of metastatic disease, tumor androgen sensitivity, patient comorbidities, prior treatments, and patient goals to guide shared decision-making. Historically, MPC has been treated primarily with palliative systemic therapies, while local therapy using either radiation or surgery has been reserved for symptom management. More recently, there is growing evidence that definitive-intent local therapy to the primary tumor and metastatic sites may improve outcomes, with the bulk of data supporting this approach in the setting of patients with low metastatic burden (LMB), specifically oligometastasis. In this manuscript, we review the literature on contemporary systemic therapies and the use of local therapy directed to the primary tumor and metastases to optimize the therapeutic index in the management of MPC.

## 2. Defining Low Metastatic Burden in MPC

The primary definition of LMB and high metastatic burden (HMB), as used to guide systemic and local therapy decisions, is based on the CHAARTED trial. LMB is typically defined as the absence of visceral metastases, <4 bone metastases or any number of bone metastases that are confined to the axial skeleton (spine and pelvis), and any number of lymph node metastases. HMB is defined as ≥4 bone metastases, with at least one outside of the axial skeleton, or the presence of visceral metastases [3]. Of note, this definition was based on the presence of metastases on conventional imaging (CT and bone scan), leading to some degree of uncertainty in defining these two groups in the PSMA PET era.

## 3. Defining Oligometastasis in MPC

Oligometastasis was initially defined by Weichselbaum and Hellman over three decades ago as “a state of cancer in which metastases are limited in number and location before the tumor becomes overtly Metastatic [4].” It is usually defined as cancer with up to three to five metastatic sites with or without active cancer at the primary tumor site [5]. Oligometastatic cancer can be classified as either synchronous, or “de novo,” when it is identified at the time of initial diagnostic work-up, versus metachronous, when it is identified after one or more courses of treatment. Metachronous oligometastasis can be further described as oligorecurrent, when tumors appear during surveillance after curative therapy, or oligoprogressive, when few tumors progress while the others remain controlled during a course of systemic therapy.

## 4. The Benefits and Challenges of PSMA PET/CT in Identifying MPC

The evolving definitions of oligometastatic prostate cancer are complemented by major advances in the detection of metastatic disease. PSMA PET/CT scans employ radiotracers with a ligand that binds to PSMA, a transmembrane protein that is overexpressed in prostate cancer cells [6]. A recent prospective trial by Lengana et al. showed higher sensitivity (96% vs. 73%) and accuracy (99% vs. 84%) for PSMA PET scans than the conventionally used bone scan for detecting skeletal lesions [7]. Pro-PSMA was the multicenter randomized study that demonstrated a clear edge to PSMA PET/CT over conventional imaging in detecting pelvic nodal or distant metastasis with a 27% greater accuracy (92% vs. 65%; *p* < 0.0001) [8]. ^18^F-DCFPyL (piflufolastat F 18) and ^68^Ga-PSMA-11 are radiotracers targeting PSMA, and the OSPREY study demonstrated the high positive predictive value of ^18^F-DCFPyL PET scans in both high-risk patients undergoing surgery and patients with suspected recurrent or metastatic disease on conventional imaging [9,10,11]. Similarly, CONDOR was a phase III randomized trial that showed the correct localization rate of ^18^F-DCFPyL PET of 84.7% to 87%, which led to change in the intended management of 63.9% of the evaluable patients [12]. Hope et al. studied the role of ^68^Ga-PSMA PET in biochemically recurrent prostate cancer, and they revealed that ^68^Ga-PSMA PET led to change in management of 53% of the patients. As shown above, different radiotracers with affinity for PSMA have consistently led to improvements in the diagnosis of skeletal, lymph nodal, and visceral metastasis and have also prompted changes in management for significant numbers of patients [13]. In a multicenter retrospective review by Onal et al., they found significantly higher prostate cancer-specific survival in patients staged with PSMA PET scans when compared against patients staged with conventional imaging (95.1% vs. 76.9%, *p* = 0.01) for node-positive prostate cancer patients that underwent definitive radiation to the prostate and pelvis [14].

The improved sensitivity of PSMA PET over conventional imaging leads to significant stage migration, where, for example, patients who would have previously been classified as having localized prostate cancer are instead found to have metastatic disease. As a result, patients of any stage based on conventional imaging may in fact have more advanced disease, on average, than patients of the same stage based on PSMA PET. This raises questions about how to adapt clinical practice to account for improved staging. For example, in a patient with localized disease on conventional imaging but low-volume metastatic disease on PSMA PET, is definitive local therapy still reasonable, or should the backbone of management be systemic therapy? If a patient has oligometastasis based on conventional imaging, but additional metastasis are identified on PSMA PET/CT such that the patient falls into the HMB, should management be adjusted? When applying practices based on clinical trials that used conventional imaging, it is critical to consider that those patients could have been under-staged by contemporary standards in the PSMA PET era.

## 5. Modern Systemic Therapies in Oligometastatic and LMB Prostate Cancer

First-line systemic therapy for MPC has evolved significantly. For half a century, androgen deprivation therapy (ADT) monotherapy was the backbone of systemic therapy, regardless of metastatic disease burden. In the present, many patients benefit from doublet or triplet drug combinations that include ADT.

### 5.1. Continuous vs. Intermittent ADT

ADT was historically delivered continuously, however, investigation have explored whether an intermittent approach could better balance the benefits of ADT with the negative impact on quality of life. The SWOG 9346 trial randomized metastatic hormone-sensitive prostate cancer (HSPC) patients to receive either intermittent or continuous ADT; the drugs used were luteinizing hormone-releasing hormone analog (LHRHa) along with an anti-androgen. In total, 1535 patients were analyzed after median 9.8 years of follow-up. No significant survival difference was seen, but the continuous therapy group had a numerically higher overall survival (OS) of 5.8 years compared to the intermittent therapy group at 5.1 years. In addition, there was no significant difference in adverse events between the two arms. The authors believed that the results were inconclusive due to very few adverse events in both the groups, and that the inferiority of intermittent ADT could not be ruled out [15]. Da Silva et al. completed a separate study, where 1045 patients with locally advanced or metastatic prostate cancer were randomized to either intermittent or continuous ADT, using LHRHa along with cyproterone acetate. They found no significant difference in OS. Sexual function, however, was superior in the intermittent ADT arm [16]. Another impactful study was the trial by Crook et al., which compared intermittent versus continuous ADT for biochemically recurrent prostate cancer. The drugs used were LHRHa along with an antiandrogen. A total of 1386 patients were randomized, with no difference in OS between the two groups (8.8 vs. 9.1 years, HR = 1.02). The study noted that adverse effects in terms of sexual function, hot flashes, and urinary symptoms were significantly better in the intermittent therapy group [17]. In this study, all patients had non-metastatic biochemical recurrence using conventional imaging; however, many patients may have had occult metastatic disease that could have been identified on PSMA PET/CT. In a meta-analysis by Magnan et al., which evaluated efficacy and tolerability of intermittent vs. continuous ADT for any stage of prostate cancer, there was no difference in OS, cancer-specific survival (CSS), or progression-free survival (PFS) between the two groups. Physical and sexual functioning was also noted to be better with intermittent ADT in most of the reviewed trials [18]. Intermittent and continuous ADT both appear to be reasonable in treating MPC.

### 5.2. ADT Combined with Androgen Receptor Pathway Inhibitor (ARPI)

In the last decade, several definitive phase III randomized trials have demonstrated that the best overall survival in metastatic HSPC to date is achieved when combining ADT with Androgen Receptor Pathway Inhibitors (ARPIs). The ENZAMET trial was a prospective randomized trial of 1125 men assigned to either ADT + enzalutamide or ADT + first-generation nonsteroidal anti-androgen therapy. ADT entailed either LHRHa or surgical castration. Prostate-specific antigen (PSA)-related PFS and clinical PFS were both significantly improved in the enzalutamide group (*p* = 0.001). OS was also significantly improved in the enzalutamide group (80% vs. 72%, *p* = 0.002). With regard to adverse events, 1% of the patients receiving enzalutamide experienced seizures, and more patients reported fatigue in the enzalutamide group [19]. In the TITAN trial, a study by Chi et al., the investigators randomized metastatic HSPC patients to receive ADT in the form of LHRHa with or without apalutamide. In total, 37.3% of patients had low-volume disease. At a follow-up of 24 months, radiographic PFS was significantly better in the apalutamide group (68% vs. 47%; *p* < 0.001). Similarly, OS was improved in the apalutamide arm (82.4% vs. 73.5%, *p* = 0.005). A total of 42.2% of patients in the apalutamide group had grade 3 or 4 adverse events compared to 40.8% in the control group [20]. The ARCHES study evaluated the addition of enzalutamide (vs. placebo) to ADT in patients with HSPC. ADT was in the form of either LHRHa or surgical castration. Patients in the enzalutamide arm had significantly reduced risk of metastatic progression or death compared to the placebo arm. The benefit was demonstrated in all prespecified subgroups, including low-volume and high-volume metastatic disease [21]. A post hoc analysis of this trial was performed by Armstrong et al., stratifying patients into oligometastatic (one to five lesions) and polymetastatic patients. The results revealed that ADT combined with enzalutamide improved radiographic PFS (*p* < 0.001), OS (*p* < 0.001), radiographic PFS (*p* < 0.001), and OS (*p* = 0.004) for both polymetastatic and oligometastatic patients [22]. Two sub-studies of the multi-arm, multistage STAMPEDE study randomized both metastatic and very-high-risk non-metastatic patients to receive ADT with or without abiraterone and prednisolone. ADT was either LHRHa, luteinizing hormone-releasing hormone antagonist, or surgical castration. In total, 52% of the enrolled patients had metastatic disease, and overall, there were significantly fewer deaths in the ADT + abiraterone group (Hazard Ratio 0.63; *p* < 0.001) compared to the ADT-alone group, but with a higher rate of grade 3 to 5 adverse events (47% vs. 33%) [23]. Finally, the LATITUDE study enrolled high-risk metastatic patients (two or more of the following three features: Gleason ≥ 8, three or more visible lesions on the bone scan, or the presence of measurable visceral metastasis not inclusive of lymph node metastasis) to be randomized to ADT with or without abiraterone and prednisone. Androgen deprivation was in the form of LHRHa or surgical castration. At a median follow-up of 51.8 months, OS was significantly better in the abiraterone and prednisone arm (53.3 months vs. 36.5 months; *p* < 0.0001) [24].

Zhang et al. recently published a meta-analysis discussing systemic treatment modalities for metastatic castration-sensitive prostate cancer [25]. In their review, they analyzed 8704 patients from seven different studies that compared the effectiveness of four ADT combination treatments, including ADT + docetaxel, ADT + abiraterone acetate, ADT + enzalutamide, and ADT + apalutamide, compared to ADT alone. The seven studies analyzed are mentioned in the above and below paragraphs (GETUG-AFU15, CHAARTED, STAMPEDE, LATITUDE, ARCHES, ENZAMET, and TITAN). This meta-analysis concluded that combination therapy of ADT + ARPI had better outcomes with regard to OS and PFS compared to ADT alone or ADT + docetaxel. Their review emphasized the importance of an individualized, patient-specific approach to determining treatment strategies, with therapeutic decisions being driven based on initial diagnostic characteristics.

### 5.3. ADT Combined with Chemotherapy

Higher metastatic burden may be associated with greater benefit of certain systemic therapy strategies, such as the use of docetaxel in HSPC. The CHAARTED trial was a phase III randomized trial that enrolled 790 patients with metastatic HSPC, stratified as either LMB or HMB, to receive ADT with or without docetaxel. The study found that OS was significantly better for patients receiving docetaxel who had HMB disease (51.2 months vs. 34.4 months; *p* < 0.001), but not for those with LMB disease (*p* = 0.86) [26]. The STAMPEDE trial also included a phase III arm that randomized HSPC patients to standard of care (SOC) vs. SOC + docetaxel. After a median follow-up of 78.2 months the study revealed that docetaxel improves OS (HR = 0.81, 95% CI 0.69–0.95, *p* = 0.009) and PFS (*p* < 0.001) regardless of metastatic burden [27]. GETUG-AFU15 was another phase III randomized trial that evaluated metastatic HSPC patients to receive ADT alone vs. ADT + docetaxel. In patients with HMB, OS showed a non-significant trend toward improvement with docetaxel (39.8 months vs. 35.1 months), while there was no improvement in survival for patients with LMB [28]. Vale et al. conducted a meta-analysis of the above three studies and revealed that docetaxel improves OS (*p* < 0.0001), PFS (*p* < 0.0001), and failure-free survival (FFS) (*p* < 0.001). However, there was no significant improvement for the subgroup with LMB [29]. While the ADT + docetaxel doublet may not outperform combinations of ADT + ARPI per the above-mentioned meta-analysis by Zhang et al., docetaxel may still have a role in treating patients with HSPC with HMB.

Building on these results, recent trials (PEACE-1, ARASENS) have explored “triplet” combinations of ADT + ARPI + docetaxel with favorable outcomes. In PEACE-1, the combination of ADT + abiraterone + docetaxel showed an improved OS of 5.1 years vs. 3.5 yrs with ADT + abiraterone in patients with HMB. Meanwhile, the ARASENS trial showed that darolutamide added to ADT and docetaxel improved OS (HR 0.68, 95% CI 0.57–0.80), with benefits observed across the trial population. As such, guidelines now include a recommendation for triplet therapy in patients with de novo high-volume metastatic HSPC who are fit for chemotherapy. With a variety of systemic therapy options, treatment should be individualized by taking into account disease characteristics, patient factors, and overall treatment goals [30,31].

## 6. Local Therapy in Metastatic Prostate Cancer

Prostate-directed therapy should be considered in select patients with metastatic prostate cancer, with the theoretical benefits of reducing tumor burden and slowing the development of new metastasis. Ongoing trials are exploring both prostatectomy and radiotherapy in this setting.

### 6.1. Prostatectomy in MPC

In the clinical literature, prostatectomy in MPC is most often proposed in the setting of LMB or for the management of significant local symptoms. A study by Cifuentes et al. using a mouse model for MPC revealed that removing the primary tumor slowed the progression of metastatic disease [32]. A literature review by Metcalfe et al. for the role of Radical Prostatectomy (RP) in metastatic prostate cancer showed that RP is a feasible procedure and may improve outcomes in patients with metastatic prostate cancer [33]. A meta-analysis of nine retrospective studies including 36,947 patients, conducted by Wang et al., found that the use of RP in MPC was associated with increased OS (pooled HR = 0.49, 95% CI = 0.44–0.55) and decreased cancer-specific mortality (pooled HR = 0.41, 95% CI = 0.36–0.47) [34]. Heidenreich et al. prospectively evaluated the role of RP in patients with low-volume skeletal metastasis, which they defined as having three or fewer skeletal metastases on bone scans and an absence of visceral metastasis. In the study, RP was performed after the PSA decreased to less than 1 ng/mL subsequent to neoadjuvant ADT. They observed that patients who underwent RP, when compared to a retrospective control cohort, had significantly improved PFS and CSS rates (38 vs. 26 months, *p* = 0.032 and 95 vs. 84%, *p* = 0.043). The complication rate was 17.4% for grade one adverse events, 8.7% for grade two, and 13% for grade three, and there were no grade four or five complications [35]. In a prospective cohort study by Poelaert et al., they evaluated safety and early local symptoms in patients with MPC who underwent RP. Forty-six patients were enrolled, out of which 17 underwent surgery. Seven patients had grade 1–2 complications postoperatively in the first 3 months. Five patients had stress incontinence after surgery, but overall local urinary symptoms were worse in patients receiving standard of care without RP, due to urge incontinence and obstructive urinary symptoms. The authors concluded that RP is safe, with a lower likelihood of local symptoms after surgery [36]. On the other hand, a phase 1 study by Yuh et al. evaluating cytoreductive prostatectomy in MPC observed some worrisome complications. Of the thirty-two patients with MPC who underwent surgery, two patients (6.25%) had major complications in the first 90 days, including one death. So, the conclusion was to limit cytoreductive surgery to clinical trials [37]. In summary, there remains a lack of randomized trials evaluating the role of cytoreductive RP in MPC patients, and it remains unclear which patients with MPC might benefit. SWOG 1802 is an ongoing phase 3 randomized controlled trial that may address this gap in evidence. It is randomizing patients to definitive local treatment (with surgery or radiation) vs. systemic therapy alone after an initial 22–28 weeks of systemic therapy for MPC. Study completion is estimated for 2031.

### 6.2. Prostate-Directed Radiotherapy in MPC

Traditionally, prostate-directed radiotherapy in MPC has been reserved for palliation of local symptoms; however, there is growing evidence supporting the use of local therapy with higher radiation doses comparable to the curative doses used for non-metastatic disease. This has been explored in multiple large randomized trials, with particularly promising benefits for patients with low metastatic burden. These studies include STAMPEDE, HORRAD, the subsequent STOPCAP meta-analysis of both STAMPEDE and HORRAD, and PEACE1. The STAMPEDE trial’s Arm H randomized more than 2000 patients to standard-of-care treatment with or without radiation to the primary tumor. The dose and fractionation employed was either 36 Gy in 6 Gy once-weekly fractions or 55 Gy in 20 daily fractions over 4 weeks. About 42% of patients had LMB. Long-term results after a median follow-up of 61.2 months revealed that radiation to the prostate significantly improved OS in LMB patients (HR = 0·64, 95% CI 0.52, 0.79, *p* < 0.001), but not in HMB patients. The global quality of life (QoL) was similar in both groups, and there was no difference in long-term urinary toxicity of grade three or higher [38]. HORRAD was a multicenter trial including 432 patients with metastatic prostate cancer who were randomized to receive radiation to the prostate plus standard of care versus standard of care alone. HORRAD differed from STAMPEDE in that it enrolled patients with any number of bone metastases and did not stratify patients per the LMB or the HMB criteria. The HORRAD trial found no difference in OS between the two groups but found that the time to PSA progression was significantly longer in the RT group (HR = 0.78; 95% CI: 0.63–0.97) [39]. Subsequently, the STOPCAP meta-analysis was conducted by Burdett et al., which analyzed the combined outcome of the two trials (HORRAD and STAMPEDE). STOPCAP demonstrated no OS benefit of radiation to the prostate among the entire trial population; however, when stratifying patients based on disease burden, there was a 7% OS benefit at three years in patients with less than five bone metastases who received RT to the prostate (*p* = 0.007) [40]. More recently, the PEACE1 randomized trial reported its results. The study had four distinct arms, including SOC alone, SOC + RT, SOC + abiraterone, and SOC + RT + abiraterone. The addition of radiation alone did not improve overall survival, but the addition of radiation and abiraterone to standard of care improved radiographic PFS for patients with low-volume metastatic disease (HR 0·65 [99·9% CI 0·36–1·19]; *p* = 0·019). Radiation did not appear to increase toxicity in this study and instead lowered the incidence of serious genitourinary (GU) events [31]. In totality, these studies indicate that, for men with newly diagnosed MPC with LMB, prostate RT is the standard of care as it offers OS benefit. On the other hand, in men with MPC with HMB, radiation may reduce serious adverse GU events without an associated benefit in survival outcomes.

## 7. Metastasis-Directed Therapy (MDT)

In parallel with the aforementioned trials exploring the role of prostate-directed therapy in MPC, there is emerging data on metastasis-directed therapy (MDT) in patients with oligometastatic disease, with multiple contemporary studies producing promising results. The proposed mechanisms for the benefit of MDT include prevention of both morbidity from metastatic tumors and the development of future metastases, which would stem from clonogens in the treated tumors.

Surgical resection of metastases in prostate cancer has very limited data. A narrative review of some case reports and case series by Rajwa et al. evaluating the role of surgical metastatectomy in prostate cancer revealed that surgical MDT in select sites is both safe and feasible. Importantly, the resectability of metastatic sites varies widely; lymph nodes and peripheral lung and liver lesions may be favorable sites while axial skeletal lesions would require prohibitively morbid surgeries [41]. The STOMP trial, discussed in more detail below, allowed patients to undergo surgery as MDT, but only six of the thirty-two patients assigned to MDT underwent surgery, with one pelvic lymph nodal metastases and one lung metastasis resected. Overall, surgery may be useful for MDT if there is concern for radiation toxicity and the lesion is amenable to surgery [42]. Larger studies are needed to more clearly define the potential role of metastatectomy in patients with prostate cancer.

Radiation has comparatively stronger evidence as a modality for MDT, most often in the form of stereotactic-body radiation therapy (SBRT) for patients with oligometastatic or LMB prostate cancer. SABR-COMET, an early contributor to this literature, was a phase II randomized controlled trial that enrolled 99 patients with a controlled primary tumor and one to five metastases to either SOC or SOC with MDT using SBRT. The trial enrolled patients with various primary tumors; 16 patients had primary prostate cancer. The long-term results revealed that 5-year OS was significantly better at 42.3% with MDT vs. only 17.3% for the SOC-alone arm (*p* = 0.001) [43].

As demonstrated by SABR-COMET, SBRT is an appealing modality for MDT as it employs non-invasive ablative treatments with modest and predictable toxicity. Subsequent trials have since explored the implementation of SBRT in MPC, across the settings of hormone-sensitive and castrate-resistant disease, with or without concurrent systemic therapies.

ORIOLE was a phase 2 randomized trial by Phillips et al. that enrolled 54 patients treated with SBRT for 1–3 metastasis detected by conventional imaging. Median PFS was significantly improved in patients treated with SBRT vs. Observation (not reached vs. 5.8 months, *p* = 0.002). Post hoc analysis of this trial demonstrated significant PFS advantage for patients who had all PSMA-avid lesions treated with SBRT: not reached vs. 11.8 months for those who had untreated PET-positive lesions (*p* = 0.006) [44]. This supports the potential for PSMA PET/CT to more accurately select sites for ablation through its improved sensitivity.

### 7.1. Using MDT Alone to Delay Systemic Therapy

One appealing way to use SBRT in MPC is to delay progression of disease, giving patients more time before starting hormonal therapies with their consequent side effects. STOMP was a phase II trial that recruited 62 patients with oligorecurrent MPC after previous curative intent treatment, with up to three metastatic sites, and randomized them to either MDT or surveillance. The 5-year ADT-free survival was better for the MDT group (34% vs. 8%) than the surveillance group (HR—0.57; 80% CI: 0.38–0.84, *p* value = 0.06). There was no significant difference in CRPC-free survival between the two groups [42].

OPERATIC (Oligometastatic Prostate Cancer RT Augmenting T Cells) was a phase II single arm trial evaluating the role of SBRT in patients displaying CRPC with three or fewer metastases. At two years, OS was 80%, and biochemical progression-free survival was 21%. No patients had grade three or higher adverse events [45].

Subsequently, a meta-analysis by Miszczyk et al. evaluated 22 prospective studies including 1137 patients, with a major focus on efficacy and safety of MDT. They estimated that two-year ADT-free survival is 55% for patients treated with MDT. Local control (LC) of treated tumors and OS were both 97% for patients treated with MDT. Only 0.3% of patients had grade three or higher toxicity [46]. In conclusion, the addition of MDT with SBRT allows patients to delay initiating ADT with excellent local control and low toxicity.

### 7.2. Combining MDT with Standard Systemic Therapy

While the aforementioned STOMP and ORIOLE trials reserved ADT for progression, other trials have explored the combination of upfront ADT with SBRT, radiating gross oligometastatic disease while simultaneously addressing systemic microscopic disease. The SOLAR and SATURN trials were both single-arm phase II trials that evaluated the efficacy of combining ARPI, MDT with SBRT, and 6 months of ADT, with the primary endpoint of durable PSA control ≥6 months after testosterone recovery. The primary endpoints for SOLAR, which enrolled de novo oligometastatic patients, and SATURN, which enrolled metachronous oligometastatic patients, were met in 83% and 50% of patients, respectively. Toxicity from MDT was minimal [47,48].

Furthermore, randomized trials have explored the combination of ADT and upfront MDT with SBRT. RADIOSA was a phase II randomized study of 105 patients that evaluated SBRT in patients with up to three oligorecurrent sites, with or without 6 months of ADT. SBRT + ADT had significantly better OS (32.2 months) compared to SBRT alone (15.1 months) (*p* = 0.0010). Treatment was relatively well tolerated, with only one grade three GU adverse event in the SBRT with ADT arm [49]. Although this study was not powered for OS, these findings discourage the omission of ADT in patients receiving MDT, as was previously performed in STOMP and ORIOLE.

EXTEND is another phase II trial enrolling patients with MPC who have five or fewer bone metastases and randomizing them to intermittent hormone treatment with or without MDT utilizing SBRT. PFS was the primary endpoint, and it was improved with the combined treatment (not reached vs. 15.8 months; *p* < 0.001) [50]. Another multi-institutional analysis of 263 oligometastatic HSPC patients by Deek et al., reporting outcomes of MDT alone vs. MDT + ADT, revealed that five-year combined biochemical and distant PFS was 9% vs. 19%, favoring MDT + ADT. On multivariable analysis, the use of ADT significantly improved PFS (HR 0.44, *p* < 0.001) [51].

### 7.3. MDT in the Setting of Castrate-Resistant Prostate Cancer (CRPC)

While the above studies explored MDT in the specific setting of HSPC, there are similarly designed phase II trials demonstrating the efficacy of MDT in CRPC. ARTO was a randomized phase II trial that enrolled patients with CRPC to evaluate the addition of MDT with SBRT to standard ADT, abiraterone acetate, and prednisone. The primary end point of biochemical response was significantly improved with MDT (92% vs. 68.3%, *p* = 0.001). After a median follow-up of 24.9 months, PFS was also significantly improved in the MDT arm: not reached in the MDT arm vs. 17 months in the control arm (hazard ratio 0.35, *p* < 0.001) [52].

More recently, the outcomes of the PCS-9 study by Niazi et al. were published. PCS-9 is a phase 2 trial that randomized patients with oligometastatic CRPC to ADT + enzalutamide with or without SBRT to metastatic sites. The SBRT arm had significantly improved median radiographic PFS (4.6 years vs. 2.3 years, *p* = 0.014). No grade 4 or 5 toxicities were reported in the SBRT arm [53]. In summary, these phase II trials demonstrate impressive PFS when combining MDT with modern systemic therapies in oligometastatic CRPC.

### 7.4. MDT with Radiopharmaceuticals

Radiopharmaceuticals have a growing role in the treatment of MPC, including in combination with MDT. The results of the LUNAR trial, which combined Lu177 radioligand therapy with SBRT, were recently published. In this study, 92 patients with oligometastatic HSPC were randomized to SBRT to metastatic sites vs. two cycles of neoadjuvant Lu177 followed by SBRT to metastatic sites. The primary end point was PFS, and it was significantly improved with the combination therapy from 7.4 months to 17.6 months (HR 0.37, *p* < 0.0001). Grade 3 hematologic adverse events were observed in 6.7% of patients in the combination arm vs. 4.8% in the SBRT-alone arm [54]. Combining SBRT with Ra-223 has been less successful. In the phase 2 RAVENS trial by Wang et al., SBRT for oligometastatic HSPC, with or without Ra-223, was studied in 64 patients to understand if the addition of beta emitter Ra-223 could delay progression. The PFS was 11.8 months with SBRT vs. 10.5 months with SBRT combined with Ra-223, and the difference was not significant [55].

These aforementioned studies in oligometastatic prostate cancer (OMPC) use a variety of strategies including surveillance, systemic therapy alone, systemic therapy with MDT, and MDT alone. This wide range of options highlights the equipoise held by practitioners regarding the management of OMPS. While several trials employing MDT are encouragingly positive for endpoints such as biochemical response, ADT-free survival, and PFS, these benefits must be weighed against the risk of excess toxicity. Furthermore, the evidence base surrounding MDT in OMPC should be interpreted with caution as it lacks phase III trials evaluating overall survival.

## 8. Salvage Therapy for Regional Nodal Oligorecurrent Prostate Cancer

In the de novo setting, pelvic node-positive prostate cancer is often treated with curative intent, and, similarly, pelvic nodal oligorecurrence may be treated with definitive salvage therapy [56]. While the above trials might support MDT with SBRT in this oligorecurrent setting, regional control may be improved by instead using conventionally fractionated radiotherapy with elective nodal irradiation (ENRT) targeting potential microscopic disease in neighboring pelvic lymph nodes. The PEACE-V STORM trial enrolled 196 patients with up to five PET-positive recurrent pelvic nodes and randomized to SBRT with 30 Gy in three fractions versus elective nodal irradiation with 45 Gy to the pelvic elective volume and 65 Gy via simultaneous-integrated boost to involved lymph nodes. All patients received 6 months of ADT. Four-year metastasis-free survival was 63% in the MDT group versus 76% in the ENRT group (*p* = 0·063) [57]. Although this difference was not significant, these findings demonstrate that ENRT may be the preferred approach in this setting.

## 9. PSMA Radioligand Therapy

Lutetium 177 vipivotide tetraxetan is a radioligand therapy that has been integrated into PSMA-positive MPC. It works by targeting and destroying cancer cells that express PSMA throughout the body. The VISION trial by Sartor et al. randomized 831 patients with metastatic CRPC who had received one ARPI and one or two taxane-based regimens to Pluvicto + SOC versus SOC alone. The results revealed that both PFS (8.7 months vs. 3.4 months; *p* < 0.001) and OS (15.3 vs. 11.3 months) were significantly improved in the Pluvicto arm. In total, 52.7% of patients in the Pluvicto arm had greater than grade two side effects versus 38% in the SOC arm [58]. The THERAP trial by Hofman et al. compared Pluvicto vs. cabazitaxel for metastatic CRPC patients who progressed after docetaxel. It was a phase II study evaluating 200 patients and demonstrated a higher response rate with fewer high-grade toxicities for Pluvicto versus cabazitaxel, although there was no significant difference between OS (19.1 vs. 19.5 months; *p* = 0.77) [59]. Mader et al. retrospectively studied the role of intermittent therapy with Pluvicto in 19 patients with oligometastatic CRPC. The researchers discontinued Pluvicto after 2 cycles if the patients responded and then resumed if disease progressed. The median OS was 45 months (95% CI to be 28–62 months) and median PFS was 27 months (95% CI to be 23 to 31 months) [60]. Theranostics such as Lutetium-177 vipivotide tetraxetan are an emerging cancer care strategy that integrates diagnostic imaging with targeted therapy, using specific probes to customize and monitor radionuclide treatments. Research has focused on utilizing 99m Tc-MDP and 18-F-NaF scans before administering Ra-223 therapy. In a similar vein, pairing PSMA ligands with PET imaging enhances patient selection for radioligand therapy by providing vital data on tumor biology. While PET-based radiomics holds significant promise for advancing prostate cancer management, this modality is new and needs further exploration [61].

## 10. Conclusions

As metastases from prostate cancer are more readily detected when incorporating PSMA PET imaging, it becomes increasingly pertinent to understand the various treatment paradigms of MPC in the modern era. ADT remains a standard backbone of therapy, with a growing number of additional systemic therapies to further improve outcomes. Current guidelines recommend triplet therapy for fit patients with de novo high-volume metastatic HSPC, while other patients should be treated with ADT + ARPI. Because multiple excellent systemic options exist, clinicians should individualize treatment based on specific disease traits, patient health, and therapeutic objectives. For LMB prostate cancer patients, many emerging studies focus on more aggressive local therapy. Radiotherapy to the primary prostate tumor is now a standard first-line treatment option for newly diagnosed low-volume metastatic disease, as it has demonstrated survival benefit, prolongation of disease control, and improvement in local symptoms without significant complications. RP may be considered for selected patients to prevent local symptomatic complications (e.g., bleeding or blockage), but it has not yet proven to be equivalent to radiation for LMB prostate cancer. Evidence for MDT is becoming stronger for both HSPC and CRPC patients, particularly in the oligometastatic setting, and primarily using radiation. MDT may delay progression and allow clinicians to de-intensify, or at least delay, systemic treatment. Incorporation of PSMA PET in radiation planning appears promising. Combination of radioligand therapy along with SBRT for metastasis has shown some promising early results; however, it remains investigational at this time. As the aforementioned studies demonstrate that multiple different approaches to treating oligometastatic HSPC and CRPC have shown benefits, the optimal treatment for each patient should be individualized with discussions surrounding disease status, the patient’s overall health and performance status, side effects of different modalities, and patient preference. Table 1 provides a brief summary of recommended treatment options for different clinical scenarios.

## 11. Future Directions and Research

**Figure 1:**
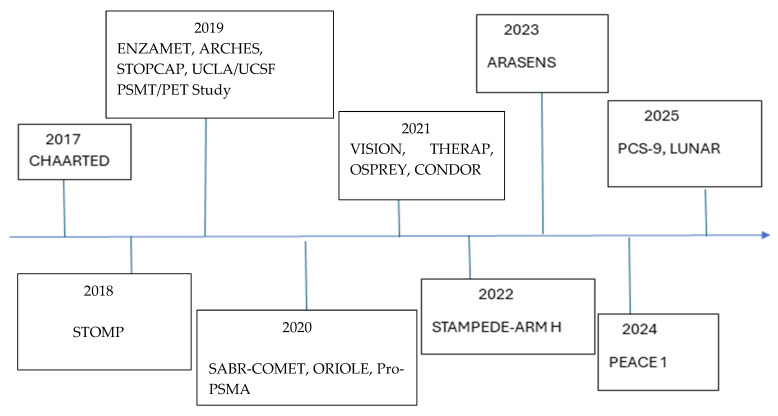
Timeline of major trials for metastatic prostate cancer.

The timeline of seminal studies in prostate cancer is depicted in Figure 1. There are many ongoing prospective studies evaluating the role of MDT in oligometastatic prostate cancer in both HSPC and CRPC. Important trials are summarized in Table 2. Future research in OMPC should prioritize leveraging the high sensitivity of PSMA-PET to personalize treatment and enhance long-term survival. As researchers work to refine the definitions of “low metastatic burden” in the PSMA-PET era, upcoming studies employ more nuanced criteria than the simple number of metastatic lesions, such as tumor biology and other clinical factors. Advanced analytical methods like artificial intelligence are being researched to enhance image interpretation, improve specificity, and differentiate malignant from benign findings. Ongoing research is also focusing on using PSMA-PET-guided MDT to delay initiation of ADT and reduce treatment-related toxicity. Moving beyond late-stage use, trials such as BULLSEYE are exploring 177Lu-PSMA-617 earlier in the disease course for oligometastatic patients.

## Figures and Tables

**Table 1:** Summary of treatment options.

Clinical Scenario	Systemic Therapy	Local Therapy
De novo HMB	ADT ± ARPI ± Docetaxel	- Radiation to prostate (if local symptoms present or impending) - Consider MDT if limited metastatic lesions
De novo LMB	ADT ± ARPI (preferred) or Docetaxel	- MDT to metastatic lesion
De novo oligometastatic	ADT ± ARPI (preferred) or Docetaxel	- Radiation to prostate - MDT to metastatic lesion
Oligorecurrent	- Consider ADT ± ARPI - May Delay Systemic Therapy if using MDT	- Consider MDT - If pelvis node-only recurrence, consider ENRT with MDT
Oligoprogressive	- May continue current systemic therapy if using MDT - Consider change to next-line systemic therapy	- Consider MDT

**Table 2:** Ongoing randomized trials of oligometastasis local therapy.

Number	Study Title and NCT Clinical Trials Number	Randomization	Patient Population	Primary Outcome	Estimated Completion Date
1.	VA STARPORT (NCT04787744)	Standard of care alone vs. standard of care + PET-directed local therapy	De novo or recurrent HSPC with up to 10 metastases	CRPC-free survival	September 2028
2.	STAMPEDE-2 (NCT06320067)	Effect of adding SBRT (SBRT-eligible) or Lu-177 PSMA radioligand therapy (SBRT-ineligible) to standard of care	De novo or metastatic HSPC with any number of metastases	OS	April 2031
3.	METANOVA (NCT06150417)	MDT to all sites + standard of care vs. standard of care alone	De novo oligometastatic HSPC with up to 10 metastasis	FFS	July 2027
4.	TERPS (NCT05223803)	MDT to all sites + standard of care vs. standard of care alone	De novo or recurrent HSPC with up to 5 metastasis	2 year FFS	July 2026
5.	STAR-TRAP	MDT to all sites + standard of care vs. Standard of care alone	Oligometastatic HSPC on systemic treatment with up to 5 metastasis	FFS	January 2028
6.	CORE (NCT02759783)	SBRT to metastatic sites + standard of care vs. standard of care alone	Oligometastatic breast, non-small cell lung cancer, and prostate cancer	PFS	June 2024
7.	EA8191 (INDICATE) (NCT04423211)	MDT + standard of care vs. standard of care alone for MPC and enhanced systemic therapy vs. standard of care alone for non-metastatic prostate cancer	Biochemically relapsed prostate cancer post-surgery	PFS	December 2032
8.	ADOPT (NCT04302454)	Metastasis-directed radiation alone vs. Metastatic-directed radiation + ADT	Oligometastatic HSPC with up to 4 metastases	Metastasis PFS	December 2027
9.	PERSIAN (NCT05717660)	ADT + apalutamide vs. ADT + apalutamide + SBRT to all metastatic sites	Oligometastatic HSPC with up to 5 metastases	Complete biochemical response	January 2025
10.	CCTG PR20 PLATON (NCT03784755)	MDT + standard of care vs. standard of care alone	Oligometastatic HSPC with up to 5 metastases	FFS	July 2027
11.	PEACE VI (Olig-PRESTO) (NCT06496581)	Standard of care vs. standard of care + 4 cycles of Lu-177 PSMA treatment	Metastatic HSPC with PSA > 0.2 after 6–8 months of systemic therapy initiation	OS and PFS	January 2033
12.	START MET (NCT05209243)	MDT + standard of care vs. standard of care alone	Oligometastatic HSPC with up to 5 metastases	Radiologic PFS	January 2027
13.	PILLAR (NCT03503344)	Apalutamide alone vs. apalutamide + SBRT to all metastatic sites	Oligometastatic CRPC with up to 5 metastases	Median time to PSA progression	January 2027

## Data Availability

No new data was created for this study.

## Abbreviations

ADT	Androgen deprivation therapy
ARPI	Androgen Receptor Pathway Inhibitor
CRPC	Castrate-resistant prostate cancer
CSS	Cancer-specific survival
ENRT	Elective nodal irradiation
FFS	Failure-free survival
GU	Genitourinary
HMB	High metastatic burden
HSPC	Hormone-sensitive prostate cancer
LC	Local control
LHRHa	Luteinizing hormone-releasing hormone analog
LMB	Low metastatic burden
MDT	Metastasis-directed therapy
MPC	Metastatic prostate cancer
OMPC	Oligo-metastatic prostate cancer
OS	Overall survival
PET	Positron emission tomography
PFS	Progression-free survival
PSA	Prostate-specific antigen
PSMA	Prostate-specific membrane antigen
QoL	Quality of life
RP	Radical Prostatectomy
RT	Radiation Therapy
SBRT	Stereotactic-body radiation therapy
SOC	Standard of care

## References

[1] Siegel R.L., Kratzer T.B., Giaquinto A.N., Sung H., Jemal A. (2025). Cancer Statistics, 2025. CA Cancer J. Clin..

[2] Chai Z., Yan S., Li H., Dong X., Li S., Fan Y., Dong Z., He Z., Zhou J., Lei P. (2025). Temporal trends and regional disparities in prostate cancer mortality in the United States, 1999–2023: An analysis of the CDC WONDER database. BMC Public Health.

[3] Sweeney C.J., Chen Y.-H., Carducci M., Liu G., Jarrard D.F., Eisenberger M., Wong Y.-N., Hahn N., Kohli M., Cooney M.M. (2015). Chemohormonal Therapy in Metastatic Hormone-Sensitive Prostate Cancer. N. Engl. J. Med..

[4] Weichselbaum R.R., Hellman S. (2011). Oligometastasis revisited. Nat. Rev. Clin. Oncol..

[5] Reyes D.K., Pienta K.J. (2015). The biology and treatment of oligometastatic cancer. Oncotarget.

[6] Zhao R., Li Y., Nie L., Qin K., Zhang H., Shi H. (2021). The meta-analysis of the effect of 68Ga-PSMA-PET/CT diagnosis of prostatic cancer compared with bone scan. Medicine.

[7] Lengana T., Lawal I.O., Boshomane T.G., Popoola G.O., Mokoala K.M., Moshokoa E., Maes A., Mokgoro N.P., Van de Wiele C., Vorster M. (2018). ^68^Ga-PSMA PET/CT Replacing Bone Scan in the Initial Staging of Skeletal Metastasis in Prostate Cancer: A Fait Accompli?. Clin. Genitourin. Cancer.

[8] Hofman M.S., Lawrentschuk N., Francis R.J., Tang C., Vela I., Thomas P., Rutherford N., Martin J.M., Frydenberg M., Shakher R. (2020). Prostate-specific membrane antigen PET-CT in patients with high-risk prostate cancer before curative-intent surgery or radiotherapy (proPSMA): A prospective, randomized, multicentre study. Lancet.

[9] Pienta K.J., Rowe M.A., Rowe S.P., Carroll P.R., Pouliot F., Probst S., Saperstein L., Preston M.A., Alva A.S., Patnaik A. (2021). A Phase 2/3 Prospective Multicenter Study of the Diagnostic Accuracy of Prostate Specific Membrane Antigen PET/CT with ^18^F-DCFPyL in Prostate Cancer Patients (OSPREY). J. Urol..

[10] Hope T.A., Eiber M., Armstrong W.R., Juarez R., Murthy V., Lawhn-Heath C., Behr S.C., Zhang L., Barbato F., Ceci F. (2021). Diagnostic Accuracy of 68Ga-PSMA-11 PET for Pelvic Nodal Metastasis Detection Prior to Radical Prostatectomy and Pelvic Lymph Node Dissection: A Multicenter Prospective Phase 3 Imaging Trial. JAMA Oncol..

[11] Fendler W.P., Calais J., Eiber M., Flavell R.R., Mishoe A., Feng F.Y., Nguyen H.G., Reiter R.E., Rettig M.B., Okamoto S. (2019). Assessment of 68Ga-PSMA-11 PET Accuracy in Localizing Recurrent Prostate Cancer: A Prospective Single-Arm Clinical Trial. JAMA Oncol..

[12] Morris M.J., Gorin S.P., Gorin M.A., Saperstein L., Pouliot F., Josephson D., Wong J.Y.C., Pantel A.R., Cho S.Y., Gage K.L. (2021). Diagnostic Performance of ^18^F-DCFPyL-PET/CT in Men with Biochemically Recurrent Prostate Cancer: Results from the CONDOR Phase III, Multicenter Study. Clin. Cancer Res..

[13] Hope T.A., Aggarwal R., Chee B., Tao D., Greene K.L., Cooperberg M.R., Feng F., Chang A., Ryan C.J., Small E.J. (2017). Impact of ^68^Ga-PSMA-11 PET on Management in Patients with Biochemically Recurrent Prostate Cancer. J. Nucl. Med..

[14] Onal C., Guler O.C., Erpolat P., Hurmuz P., Sutera P., Deek M.P., Elmali A., Yilmaz M.T., Koken U.H., Yavuz M. (2024). Evaluation of Treatment Outcomes of Prostate Cancer Patients with Lymph Node Metastasis Treated With Definitive Radiotherapy: Comparative Analysis of PSMA PET/CT and Conventional Imaging. Clin. Nucl. Med..

[15] Hussain M., Tangen C.M., Berry D.L., Higano C.S., Crawford E.D., Liu G., Wilding G., Prescott S., Sundaram S.K., Small E.J. (2013). Intermittent versus continuous androgen deprivation in prostate cancer. N. Engl. J. Med..

[16] da Silva F.C., da Silva F.M.C., Gonçalves F., Santos A., Kliment J., Whelan P., Oliver T., Antoniou N., Pastidis S., Queimadelos A.M. (2014). Locally advanced and metastatic prostate cancer treated with intermittent androgen monotherapy or maximal androgen blockade: Results from a randomised phase 3 study by the South European Uroncological Group. Eur. Urol..

[17] Crook J.M., O’Callaghgan C.J., Duncan G., Dearnaley D.P., Higano C.S., Horwitz E.M., Frymire E., Malone S., Chin J., Nabid A. (2012). Intermittent androgen suppression for rising PSA level after radiotherapy. N. Engl. J. Med..

[18] Magnan S., Zarychanski R., Pilote L., Bernier L., Shemilt M., Vigneault E., Fradet V., Turgeon A.F. (2015). Intermittent vs Continuous Androgen Deprivation Therapy for Prostate Cancer: A Systematic Review and Meta-analysis. JAMA Oncol..

[19] Davis I.D., Martin A.J., Stockler M.R., Begbie S., Chi K.N., Chowdhury S., Coskinas X., Frydenberg M., Hague W.E., Horvath L.G. (2019). Enzalutamide with Standard First-Line Therapy in Metastatic Prostate Cancer. N. Engl. J. Med..

[20] Chi K.N., Agarwal N., Bjartell A., Chung B.H., de Santana Gomes A.J.P., Given R., Soto Á.J., Merseburger A.S., Özgüroğlu M., Uemura H. (2019). Apalutamide for Metastatic, Castration-Sensitive Prostate Cancer. N. Engl. J. Med..

[21] Armstrong A.J., Szmulewitz R.Z., Petrylak D.P., Holzbeierlein J., Villers A., Azad A., Alcaraz A., Alekseev B., Iguchi T., Shore N.D. (2019). ARCHES: A Randomized, Phase III Study of Androgen Deprivation Therapy With Enzalutamide or Placebo in Men With Metastatic Hormone-Sensitive Prostate Cancer. J. Clin. Oncol..

[22] Armstrong A.J., Iguchi T., Azad A.A., Villers A., Alekseev B., Petrylak D.P., Szmulewitz R.Z., Alcaraz A., Shore N.D., Holzbeierlein J. (2023). The Efficacy of Enzalutamide plus Androgen Deprivation Therapy in Oligometastatic Hormone-sensitive Prostate Cancer: A Post Hoc Analysis of ARCHES. Eur. Urol..

[23] James N.D., de Bono J.S., Spears M.R., Clarke N.W., Mason M.D., Dearnaley D.P., Ritchie A.W.S., Amos C.L., Gilson C., Jones R.J. (2017). Abiraterone for Prostate Cancer Not Previously Treated with Hormone Therapy. N. Engl. J. Med..

[24] Fizazi K., Tran N., Fein L., Matsubara N., Rodriguez-Antolin A., Alekseev B.Y., Özgüroğlu M., Ye D., Feyerabend S., Protheroe A. (2019). Abiraterone acetate plus prednisone in patients with newly diagnosed high-risk metastatic castration-sensitive prostate cancer (LATITUDE): Final overall survival analysis of a randomised, double-blind, phase 3 trial. Lancet Oncol..

[25] Zhang M., Wan L., Yao Y., Wu R., Li W., Gu P. (2025). Systemic treatment of metastatic castration-sensitive prostate cancer: A meta-analysis of efficacy and safety. Medicine.

[26] Kyriakopoulos C.E., Chen Y.-H., Carducci M.A., Liu G., Jarrard D.F., Hahn N.M., Shevrin D.H., Dreicer R., Hussain M., Eisenberger M. (2018). Chemohormonal Therapy in Metastatic Hormone-Sensitive Prostate Cancer: Long-Term Survival Analysis of the Randomized Phase III E3805 CHAARTED Trial. J. Clin. Oncol..

[27] Clarke N.W., Ali A., Ingleby F.C., Hoyle A., Amos C.L., Attard G., Brawley C.D., Calvert J., Chowdhury S., Cook A. (2019). Addition of docetaxel to hormonal therapy in low- and high-burden metastatic hormone sensitive prostate cancer: Long-term survival results from the STAMPEDE trial. Ann. Oncol..

[28] Gravis G., Fizazi K., Joly F., Oudard S., Priou F., Esterni B., Latorzeff I., Delva R., Krakowski I., Laguerre B. (2013). Androgen-deprivation therapy alone or with docetaxel in non-castrate metastatic prostate cancer (GETUG-AFU 15): A randomised, open-label, phase 3 trial. Lancet Oncol..

[29] CVale L., Fisher D.J., Godolphin P.J., Rydzewska L.H., Boher J.-M., Burdett S., Chen Y.-H., Clarke N.W., Fizazi K., Gravis G. (2023). Which patients with metastatic hormone-sensitive prostate cancer benefit from docetaxel: A systematic review and meta-analysis of individual participant data from randomised trials. Lancet Oncol..

[30] Smith M.R., Hussain M., Saad F., Fizazi K., Sternberg C.N., Crawford E.D., Kopyltsov E., Park C.H., Alekseev B., Montesa-Pino Á. (2022). Darolutamide and Survival in Metastatic, Hormone-Sensitive Prostate Cancer. N. Engl. J. Med..

[31] Bossi A., Foulon S., Maldonado X., Sargos P., MacDermott R., Kelly P., Fléchon A., Tombal B., Supiot S., Berthold D. (2024). Efficacy and safety of prostate radiotherapy in de novo metastatic castration-sensitive prostate cancer (PEACE-1): A multicentre, open-label, randomised, phase 3 study with a 2 × 2 factorial design. Lancet.

[32] Cifuentes F.F., Valenzuala R.H., Contreras H.R., Castellón E.A. (2015). Surgical cytoreduction of the primary tumor reduces metastatic progression in a mouse model of prostate cancer. Oncol. Rep..

[33] Metcalfe M.J., Smaldone M.C., Uzzo R.G., Boorjian S.A., Kim S.P., Nguyen P.L., Evans C.P., Barocas D.A., Penson D.F., Karnes R.J. (2017). Role of radical prostatectomy in metastatic prostate cancer: A review. Urol. Oncol..

[34] Wang Y., Qin Z., Chen C., Wang Y., Meng X., Song N.-H. (2018). The role of radical prostatectomy for the treatment of metastatic prostate cancer: A systematic review and meta-analysis. Biosci. Rep..

[35] Heidenreich A., Pfister D., Porres D. (2015). Cytoreductive radical prostatectomy in patients with prostate cancer and low volume skeletal metastases: Results of a feasibility and case-control study. J. Urol..

[36] Poelaert F., Verbaeys C., Rappe B., Kimpe B., Billiet I., Plancke H., Decaestecker K., Fonteyne V., Buelens S., Lumen N. (2017). Cytoreductive Prostatectomy for Metastatic Prostate Cancer: First Lessons Learned From the Multicentric Prospective Local Treatment of Metastatic Prostate Cancer (LoMP) Trial. Urology.

[37] Yuh B.E., Kwon Y.S., Kawachi M.S., Pe M.W., Wilson T.G., Chan K., Ruel N., Wong J.S., Chen S.J., Lau C.S. (2019). Results of Phase 1 study on cytoreductive radical prostatectomy in men with newly diagnosed metastatic prostate cancer. Prostate Int..

[38] Parker C.C., James N.D., Brawley C.D., Clarke N.W., Ali A., Amos C.L., Attard G., Chowdhury S., Cook A., Cross W. (2022). Radiotherapy to the prostate for men with metastatic prostate cancer in the UK and Switzerland: Long-term results from the STAMPEDE randomised controlled trial. PLoS Med..

[39] Boeve L.M.S., Hulshof M.C.C.M., Verhagen P.C.M.S., Twisk J.W.R., Witjes W.P.J., de Vries P., van Moorselaar R.J.A., van Andel G., Vis A.N. (2019). Effect on Survival of Androgen Deprivation Therapy Alone Compared to Androgen Deprivation Therapy Combined with Concurrent Radiation Therapy to the Prostate in Patients with Primary Bone Metastatic Prostate Cancer in a Prospective Randomised Clinical Trial: Data from the HORRAD Trial. Eur. Urol..

[40] Burdett S., Boeve L.M., Ingleby F.C., Fisher D.J., Rydzewska L.H., Vale C.L., van Andel G., Clarke N.W., Hulshof M.C., James N.D. (2019). Prostate Radiotherapy for Metastatic Hormone-sensitive Prostate Cancer: A STOPCAP Systematic Review and Meta-analysis. Eur. Urol..

[41] Rajwa P., Yanagisawa T., Heidegger I., Zattoni F., Marra G., Soeterik T.F.W., van den Bergh R.C.N., Valerio M., Ceci F., Kesch C.V. (2023). Surgical Metastasectomy for Visceral and Bone Prostate Cancer Metastases: A Mini-Review. Eur. Urol. Focus.

[42] Ost P., Reynders D., Decaestecker K., Fonteyne V., Lumen N., De Bruycker A., Lambert B., Delrue L., Bultijnck R., Claeys T. (2018). Surveillance or Metastasis-Directed Therapy for Oligometastatic Prostate Cancer Recurrence: A Prospective, Randomized, Multicenter Phase II Trial. J. Clin. Oncol..

[43] Palma D.A., Olson R., Harrow S., Gaede S., Louie A.V., Haasbeek C., Mulroy L., Lock M., Rodrigues G.B., Yaremko B.P. (2020). Stereotactic Ablative Radiotherapy for the Comprehensive Treatment of Oligometastatic Cancers: Long-Term Results of the SABR-COMET Phase II Randomized Trial. J. Clin. Oncol..

[44] Phillips R., Shi W.Y., Deek M., Radwan N., Lim S.J., Antonarakis E.S., Rowe S.P., Ross A.E., Gorin M.A., Deville C. (2020). Outcomes of Observation vs Stereotactic Ablative Radiation for Oligometastatic Prostate Cancer: The ORIOLE Phase 2 Randomized Clinical Trial. JAMA Oncol..

[45] Zhang H., Orme J.J., Abraha F., Stish B.J., Lowe V.J., Lucien F., Tryggestad E.J., Bold M.S., Pagliaro L.C., Choo C.R. (2021). Phase II Evaluation of Stereotactic Ablative Radiotherapy (SABR) and Immunity in ^11^C-Choline-PET/CT-Identified Oligometastatic Castration-Resistant Prostate Cancer. Clin. Cancer Res..

[46] Miszczyk M., Rajwa P., Yanagisawa T., Nowicka Z., Shim S.R., Laukhtina E., Kawada T., von Deimling M., Pradere B., Rivas J.G. (2024). The Efficacy and Safety of Metastasis-directed Therapy in Patients with Prostate Cancer: A Systematic Review and Meta-analysis of Prospective Studies. Eur. Urol..

[47] Nikitas J., Rettig M., Shen J., Reiter R., Lee A., Steinberg M.L., Valle L.F., Sachdeva A., Romero T., Calais J. (2024). Systemic and Tumor-directed Therapy for Oligorecurrent Metastatic Prostate Cancer (SATURN): Primary Endpoint Results from a Phase 2 Clinical Trial. Eur. Urol..

[48] Nickols N.G., Tsai S., Kane N., Tran S., Ghayouri L., Diaz-Perez S., Thein M., Anderson-Berman N., Eason J., Kishan A.U. (2024). Systemic and Tumor-directed Therapy for Oligometastatic Prostate Cancer: The SOLAR Phase 2 Trial in De Novo Oligometastatic Prostate Cancer. Eur. Urol..

[49] Marvaso G., Corrao G., Zaffaroni M., Vincini M.G., Lorubbio C., Gandini S., Fodor C., Netti S., Zerini D., Luzzago S. (2025). ADT with SBRT versus SBRT alone for hormone-sensitive oligorecurrent prostate cancer (RADIOSA): A randomised, open-label, phase 2 clinical trial. Lancet Oncol..

[50] Tang C., Sherry A.D., Haymaker C., Bathala T., Liu S., Fellman B., Cohen L., Aparicio A., Zurita A.J., Reuben A. (2023). Addition of Metastasis-Directed Therapy to Intermittent Hormone Therapy for Oligometastatic Prostate Cancer: The EXTEND Phase 2 Randomized Clinical Trial. JAMA Oncol..

[51] Deek M.P., Sutera P., Jing Y., Gao R., Rothman E., Day H., Chang D., Dirix P., Armstrong A.J., Campbell B. (2024). Multi-institutional Analysis of Metastasis-directed Therapy with or Without Androgen Deprivation Therapy in Oligometastatic Castration-sensitive Prostate Cancer. Eur. Urol. Oncol..

[52] Francolini G., Allegra A.G., Detti B., Di Cataldo V., Caini S., Bruni A., Ingrosso G., D’Angelillo R.M., Alitto A.R., Augugliaro M. (2023). Stereotactic Body Radiation Therapy and Abiraterone Acetate for Patients Affected by Oligometastatic Castrate-Resistant Prostate Cancer: A Randomized Phase II Trial (ARTO). J. Clin. Oncol..

[53] Niazi T., Saad F., Tisseverasinghe S., Koul R., Thibault I., Chung P.W.M., Wakil G., Lock M., Delouya G., Bahoric B. (2025). Metastases-directed therapy in addition to standard systemic therapy in oligometastatic castration-resistant prostate cancer in Canada (GROUQ-PCS 9): A multicentre, open-label, randomised, phase 2 trial. Lancet Oncol..

[54] Kishan A.U., Valle L.F., Wilhalme H., Felix C., Nabong R., Juarez-Casillas J.E., Flores K., Ma T.M., Ludwig V., Nakayama M. (2025). ^177^Lu-Prostate-Specific Membrane Antigen Neoadjuvant to Stereotactic Ablative Radiotherapy for Oligorecurrent Prostate Cancer (LUNAR): An Open-Label, Randomized, Controlled, Phase II Study. J. Clin. Oncol..

[55] Wang J.H., Sherry A.D., Bazyar S., Sutera P., Radwan N., Phillips R.M., Deek M.P., Lu J., Dipasquale S., Deville C. (2025). Outcomes of Radium-223 and Stereotactic Ablative Radiotherapy Versus Stereotactic Ablative Radiotherapy for Oligometastatic Prostate Cancers: The RAVENS Phase II Randomized Trial. J. Clin. Oncol..

[56] James N.D., Spears M.R., Clarke N.W., Dearnaley D.P., Mason M.D., Parker C.C., Ritchie A.W.S., Russell J.M., Schiavone F., Attard G. (2016). Failure-Free Survival and Radiotherapy in Patients With Newly Diagnosed Nonmetastatic Prostate Cancer: Data From Patients in the Control Arm of the STAMPEDE Trial. JAMA Oncol..

[57] Ost P., Siva S., Brabrand S., Dirix P., Liefhooghe N., Otte F.-X., Gomez-Iturriaga A., Everaerts W., Shelan M., Conde-Moreno A. (2025). Salvage metastasis-directed therapy versus elective nodal radiotherapy for oligorecurrent nodal prostate cancer metastases (PEACE V-STORM): A phase 2, open-label, randomised controlled trial. Lancet Oncol..

[58] Sartor O., de Bono J., Chi K.N., Fizazi K., Herrmann K., Rahbar K., Tagawa S.T., Nordquist L.T., Vaishampayan N., El-Haddad G. (2021). Lutetium-177-PSMA-617 for Metastatic Castration-Resistant Prostate Cancer. N. Engl. J. Med..

[59] Hofman M.S., Emmett L., Sandhu S., Iravani A., Joshua A.M., Goh J.C., Pattison D.A., Tan T.H., Kirkwood I.D., Ng S. (2021). [^177^Lu]Lu-PSMA-617 versus cabazitaxel in patients with metastatic castration-resistant prostate cancer (TheraP): A randomised, open-label, phase 2 trial. Lancet.

[60] Mader N., Schoeler C., Pezeshkpour N., Klimek K., Groener D., Happel C., Tselis N., Mandel P., Grünwald F., Sabet A. (2023). Intermittent Radioligand Therapy with ^177^Lu-PSMA-617 for Oligometastatic Castration-Resistant Prostate Cancer. Cancer.

[61] Filippi L., Urso L., Bianconi F., Palumbo B., Marzola M.C., Evangelista L., Schillaci O. (2023). Radiomics and theranostics with molecular and metabolic probes in prostate cancer: Toward a personalized approach. Expert Rev. Mol. Diagn.

